# Predictive markers of depression in hypertension

**DOI:** 10.1097/MD.0000000000011768

**Published:** 2018-08-10

**Authors:** Xiuli Song, Zhong Zhang, Rui Zhang, Miye Wang, Dongtao Lin, Tao Li, Junming Shao, Xiaohong Ma

**Affiliations:** a Psychiatric Laboratory and Mental Health Center, West China Hospital of Sichuan University; b Web Sciences Center; c Big Data Research Center, University of Electronic Science and Technology of China; d Information Center, West China Hospital, Sichuan University; e College of Foreign Languages and Cultures, Sichuan University, Chengdu, PR China.

**Keywords:** classification, depression, diagnosis, hypertension, support vector machine

## Abstract

Hypertension and depression, as 2 major public health issues, are closely related. For patients having hypertension, in particular, depression is a risk factor for mortality and jeopardizes their wellbeing. The aim of the study is to apply support vector machine (SVM) learning to blood tests and vital signs to classify patients having hypertension complicated by depression and patients having hypertension alone for the identification of novel markers.

Data on patients having both hypertension and depression (n = 147) and patients having hypertension alone (n = 147) were obtained from electronic medical records of admissions containing the records on blood tests and vital signs. Using SVM, we distinguished patients having both hypertension and depression from gender- and age-matched patients having hypertension alone.

SVM-based classification achieved 73.5% accuracy by 10-fold cross-validation between patients having both hypertension and depression and those having hypertension alone. Twelve features were selected to compose the optimal feature sets, including body temperature (T), glucose (GLU), creatine kinase (CK), albumin (ALB), hydroxybutyrate dehydrogenase (HBDH), blood urea nitrogen (BUN), uric Acid (UA), creatinine (Crea), cholesterol (TC), total protein (TP), pulse (P), and respiration (R).

SVM can be used to distinguish patients having both hypertension and depression from those having hypertension alone. A significant association was identified between depression and blood tests and vital signs. This approach can be helpful for clinical diagnosis of depression, but further studies are needed to verify the role of these candidate markers for depression diagnosis.

## 1 Introduction

At present, approximately 28% of adults were diagnosed with hypertension worldwide, and the percentage was estimated to increase to 33% by 2025.^[1–3]^ Patients with hypertension usually have physical symptoms, side-effects of the antihypertensive medication, lower quality of life, possible complications of hypertension, and role impairment, which all made them vulnerable to psychological distress, especially depression.^[4–9]^

Depression accounted for 3.0% of global disability adjusted life years (DALYs) and was the second cause of disability worldwide.^[10–12]^ Prospective studies have shown that depressive symptoms were the risk factor of hypertension and that depression impaired wellbeing in patients with hypertension.^[13–15]^ A meta-analysis involving 41 studies to identify 30,796 individuals reported a 26.8% (95% CI: 21.7%–32.3%) prevalence of depression in hypertensive patients, which indicated that depression was common among patients with hypertension.^[6]^

Hypertension is the main risk factor of the occurrence and development of cerebrovascular, myocardial infarction, and congestive heart failure, and causes morbidity and mortality.^[16–18]^ Depressive symptoms and depression are also the risk factors for cardiovascular diseases.^[19]^ Previous depressive symptoms or major depressive disorders can predict a 2- to 3-fold increase in coronary heart disease and stroke disease.^[20]^ Patients who had both hypertension and depression were associated with a higher mortality and higher risk of complications.

It is well realized that depression comorbidity has a long-term negative effect on health outcomes for patients with hypertension and complications. Some proposals were made to enhance compliance among patients with heart failure by psychological interventions that may improve patient-physician cooperation, for example.^[21]^ In the real world, one significant challenge in clinical setting is the recognition of depression in patients having hypertension, which resulted in comorbidity of hypertension and depression that were undiagnosed.^[22,23]^

Recently, machine-learning-based discriminating classifications have been applied to distinguish patients with different mental disorders using individual brain images.^[24]^ An important challenge in hypertension is the early identification of individuals at risk for depression. Based on blood tests and vital signs, we examined whether SVM discriminating classification can be used to help differentiate patients with comorbidity of hypertension and depression from those with hypertension alone. This approach could help to detect the objective markers of depression in hypertension.

## 2 Materials and methods

### 2.1 Data source

Data containing the records on blood tests and vital signs were obtained from electronic medical records of admissions to West China Hospital of Sichuan University between January 1, 2011 and October 31, 2016.

### 2.2 Sample and study design

Our sample consisted of 2 groups of patients. In one group, patients were diagnosed with hypertension using the ICD-10 categories I10.x; and in the other group, patients were diagnosed with comorbidity of hypertension and depression in which depression was diagnosed according to the ICD-10 categories F32.x and F33.x. In addition, comorbidity is the common presence of 2 or more diseases, each of which meets the diagnostic criteria. The diagnosis of depression and hypertension in our study is consistent with the diagnostic criteria of ICD-10, both of which exist simultaneously. In our study, patients with other diseases and of other ethnicities rather than Han were excluded to avoid confounding effects. We extracted eligible samples for both groups from electronic information center according to the ICD-10 categories I10.x, F32.x, and F33.x. Each group sample included age, gender, race, marital status, blood tests, vital signs, expenses and so on, of which blood tests and vital signs were the first results on admission. Then we matched the age and sex of the 2 groups by using the case–control matching method in SPSS. Finally, the patients having both hypertension and depression (n = 149) and the patients having hypertension alone (n = 147) were obtained. Each department had different blood contents checked according to the needs of the diseases, so we had retained the consonant blood contents of both groups. Blood tests included red blood cell count, hemoglobin, mean corpuscular volume, mean corpuscular hemoglobin, mean corpuscular hemoglobin concentration, platelets, white blood cell count, neutrophils/100 leukocytes, lymphocytes/100 leukocytes, monocytes/100 leukocytes, eosinophils/100 leukocytes, basophils/100 leukocytes, absolute value of neutrophils, absolute value of lymphocytes, absolute value of monocytes, absolute value of eosinophils, absolute value of basophils, total bilirubin, direct bilirubin, alanine aminotransferase, bilirubin indirect, total protein, albumin (ALB), globulin (GLB), A/G, creatinine, uric acid, aspartate aminotransferase (AST), alkaline phosphatase (ALP), AST/ALT, glucose, creatine kinase, gamma-GT, lactate dehydrogenase, hydroxybutyrate dehydrogenase, triglycerides, blood urea nitrogen, cholesterol, calcium, magnesium, serum phosphorus, high-density lipoprotein, low-density lipoprotein, sodium, potassium, chlorine, and vital signs included body temperature, pulse, respiration, systolic blood pressure, and diastolic blood pressure. The study was approved by the Institutional Ethics Committee of Sichuan University and written informed consent was obtained from all participants.

### 2.3 Statistical analysis

The statistical analysis was conducted using SPSS 24.0. The normal distribution of quantitative data was presented as mean ± standard deviation. Chi-squared test and 2-sample *t* test were used for comparison between groups. Statistical significance was set at *P < .*05 for both tests.

### 2.4 Data processing

To explore whether the identified blood tests and vital signs might serve as markers for diagnosing depression, a SVM approach implemented by Weka (Waikato Environment for Knowledge Analysis [version 3.8.0]) was performed. At first, the data set was preprocessed to generate a balanced sample set, and the number of each group was 147 in the end. The results of the tested blood and vital signs were used as the features for classification. Then, we exploited the information gain-based approach to obtain the optimal feature set. To obtain an unbiased estimate of classification accuracy, we used 10-fold cross-validation to evaluate the classification performance. Namely, we used 10-fold cross-validation to evaluate the classification results. Specifically, we first randomly divided the whole data set into 10 subsets, and selected 1 set as the testing set and the other 9 sets as the training set. We used the training set to do feature selection and train SVM, and then performed classification on the testing set. The operation was repeated 10 times, and the testing and training sets were different at each time. Thus, 10 different classification results were obtained as a result of 10 times of classification. Finally, the mean value of classification results was obtained. 10-fold cross-validation relieved the error of splitting data set into training and testing sets, and each data sample was used efficiently to train the model. Note that inconsistent feature selection results could be obtained from all 10 folds. It can be solved by major vote. Finally, the performance of a classifier was assessed using the classification accuracy: (TP + TN)/(TP + TN + FP + FN), sensitivity: TP/(TP + FN), specificity: TN/(TN + FP), precision: TP/ (TP + FP) (the ratio of the actual positive patients having both hypertension and depression samples out of the predicted positive patients having both hypertension and depression samples) and recall (known as sensitivity).

## 3 Results

No significant differences were found in either sex or age between the 2 groups while significant differences were observed in total protein, albumin, creatinine, uric acid, glucose, creatine kinase, hydroxybutyrate dehydrogenase, blood urea nitrogen, cholesterol, body temperature, pulse, and respiration between the 2 groups (Table 1).

**Table 1:**
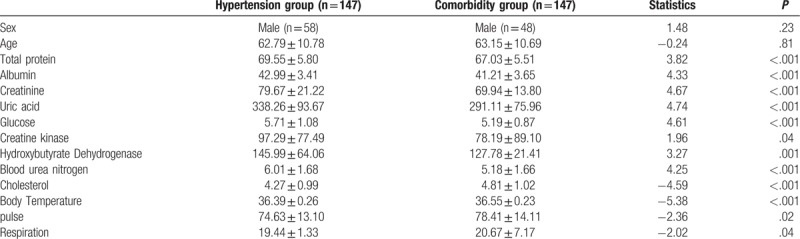
Demographic and clinical features of 294 patients having both hypertension and depression and having hypertension alone.

Among all 51 features, the following were ranked according to their importance, from high to low: body temperature (0.1084), glucose (0.0738), creatine kinase (0.0651), albumin (0.0642), hydroxybutyrate dehydrogenase (0.0546), blood urea nitrogen (0.0546), uric acid (0.0528), creatinine (0.0527), cholesterol (0.0468), total protein (0.0438), pulse (0.0393), and respiration (0.0333) (Table 2).

**Table 2:**
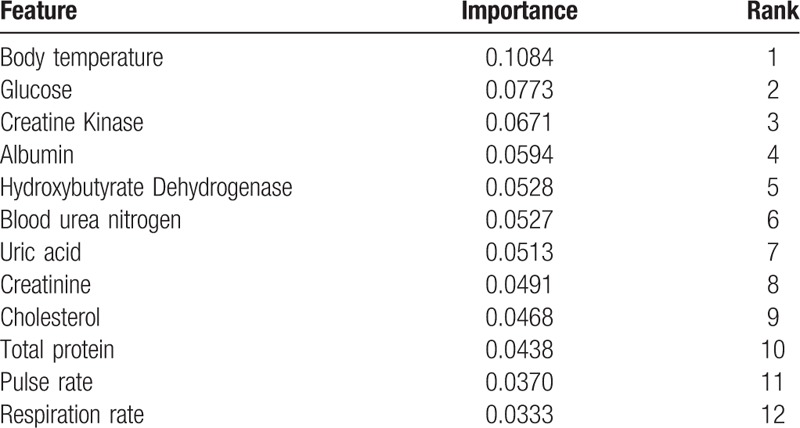
The best feature subsets of blood tests and vital signs and the rank of importance.

The classification results of 78.2% sensitivity, 68.7% specificity, and 71.4% precision, respectively, were achieved to distinguish patients with comorbidity of hypertension and depression from patients with hypertension alone (Table 3).

**Table 3:**
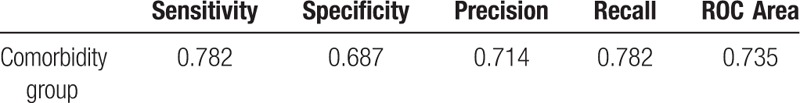
The sensitivity, specificity, and precision of recognition results.

**Figure 1:**
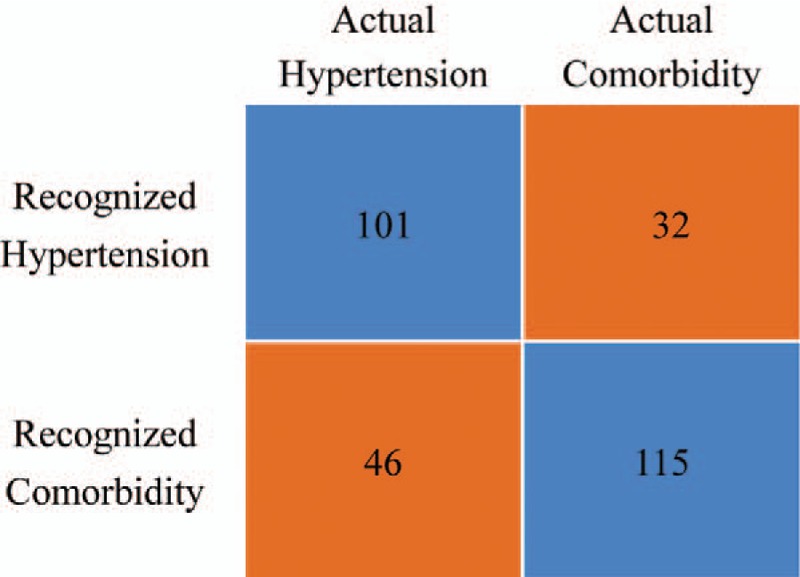
Confusion matrix. The recognition results.

There are 115 patients with comorbidity of hypertension and depression recognized from 147 patients with comorbidity of hypertension and depression (Fig. 1).

## 4 Discussion

Clinical blood tests and vital signs are routine hospital examinations for in-patients in clinical practice. We therefore combined both blood tests and vital signs using SVM to provide objective and useful information to distinguish patients with comorbidity of hypertension and depression from patients with hypertension alone. SVM is an effective classification method for combining multiple features to build a classifier. In the future, for each new patient, we put the selected markers into the trained SVM model, which can finally predict hypertensive patients with depression. We identified twelve important features relevant to depression: body temperature, glucose, creatine kinase, albumin, hydroxybutyrate dehydrogenase, blood urea nitrogen, uric acid, creatinine, cholesterol, total protein, pulse, and respiration, which differentiate patients with comorbidity of hypertension and depression from patients with hypertension at 73.5% overall classification accuracy. Features identified suggested that modulation of the metabolic substances, proteins, enzymes and nervous system were involved in the pathophysiology of depression.

The following features may have been associated with the underlying novel biological pathophysiology of depression.

### 4.1 Body temperature

Circadian rhythms include body temperature, hormone, sleep, cognitive performance, alertness and so on.^[25]^ An approximate 24-h cycle of daily physiological and biological rhythms is regulated by circadian processes. Over the past 3 decades, a large number of studies had demonstrated that various aspects of circadian rhythms were disrupted in patients having depression.^[26–28]^ One previous study found that patients having depression had a higher daytime temperature and an increased nocturnal temperature, indicating that the thermoregulatory cooling mechanisms are dysfunctional in patients with depression.^[29]^ Consistent with this, antidepressant therapy was effective in symptom relief and ameliorated illness-related circadian dysfunction.^[27]^ Researchers suggested that peripheral, serotonergic, and sensory cells were involved in relaying homeostatic environmental signals (including body temperature) to the brain, and were targets of used antidepressants, such as selective serotonin reuptake inhibitors (SSRIs). It is possible that depression involves dysregulation of both central and peripheral serotonergic systems, while successful antidepressant treatment normalizes the function of both.^[29,30]^ A large quantity of studies reported that allelic variants in tryptophan hydroxylase (TPH)1 expressed in peripheral tissue may be genetic predictors of depression^[29,31,32]^ to confirm the above hypothesis that both central and peripheral serotonergic systems were involved in the pathophysiology of depression. More recently, a longitudinal big data analysis of 35,488 patients found that body temperature can predict mortality because inflammation may lead to a higher temperature.^[33]^ To date, existing literature suggests that inflammatory markers such as C-reactive protein (CRP), interleukin-6 (IL-6), and interleukin-1 (IL-1) are positively associated with depression.^[34]^ Nevertheless, further research is needed because these factors were not examined in the present study.

### 4.2 Glucose

In the case of depression, one of its hallmarks is loss of appetite, which can affect blood sugar levels because glucose in the body is mainly derived from the ingested food. Glucose is also the obligatory energy source in the brain. Recent studies using functional magnetic resonance imaging (fMRI) and positron emission tomography (PET) showed that patients having depression presented defective glucose metabolism in brain regions including amygdala, prefrontal cortex, and hippocampus were involved in cognitive functions and emotional processing.^[35–38]^ More importantly, some beneficial effects were obtained by reversing alterations in glucose metabolism in patients having depression, particularly in the frontal limbic system by using several antidepressant treatments, including SSRIs, transcranial magnetic stimulation, cognitive-behavioral therapy, and electroconvuisive therapy (ECT).^[39–43]^ Recently, more researchers have focused on the mitochondria. In depression, there was higher mitochondrial DNA deletion, well-recorded changes in mitochondrial function, decreased blood flow, lower adenosine triphosphate (ATP) production rate, abnormal glucose metabolism, and abnormal energetic metabolism in prefrontal cortices and basal ganglia.^[44–49]^ Based on these observations, mitochondrial DNA deletion and dysfunctional glucose metabolism might also contribute to the pathophysiology of depression.

### 4.3 Creatine kinase

Serum creatine kinase (CK) is known accurate indicators of muscle damage.^[50,51]^ The normal role of CK is to catalyze the reversible transfer of the phosphoryl group from phosphocreatine to adenosine diphosphate (ADP), regenerating ATP, which is used as energy by cells.^[52]^ Some studies had reported that atypical antipsychotics and antidepressants may alter CK concentration and activity in the plasma and brain.^[53–55]^ After the antidepressant treatment with paroxetine, CK activity was increased in the prefrontal cortex, hippocampus, and striatum of rats, and CK levels were increased in the serum of a patient with depression. Thus, the increase in CK activity elevated by paroxetine may be related to increased concentrations of the enzyme. Therefore, it is important to assess the CK levels and activity, in order to evaluate the metabolic changes of depressive patients taking antidepressant drug.

### 4.4 Albumin

Serum albumin is the most abundant protein in serum—with a normal concentration of between 35 and 50 g/L and plays a crucial role in physiological and pharmacological functions.^[50,56]^ Albumin plays an important role in maintaining the osmotic pressure and transport of various endo- and exogenous ligands, as well as stabilizing the redox potential.^[56]^ In some studies, albumin was found to be related to the inflammatory response in cardiovascular disease, renal disease and uremia.^[57–59]^ Meanwhile, the relationship between albumin and depression had also been established.^[57,60]^ According to Wang et al,^[61]^ albumin level represented a poor nutritional status of patients having depression. In others studies, the serum albumin level was significantly lower in depressive patients than that in control subjects, and serum albumin levels together with IL-6 were associated with depression.^[61,62]^ Huang and Lee^[57]^ suggested that a more severe inflammatory response and greater oxidative damage were present in chronic hemodialysis patients with depression than those without. Moreover, the antidepressant treatment might lead to an increase in the levels of these antioxidants, including albumin, and uric acid.^[62,63]^ A prospective study is required to determine the causal relationships involved in psycho-neuro-immune mechanisms.

### 4.5 Hydroxybutyrate dehydrogenase (HBDH)

In recent years, researchers have begun to pay more attention to mitochondrial dysfunction as a potential pathogenic event in depression.^[64]^ In addition, studies had demonstrated increased brain lactate levels in adult patients with depression compared to healthy controls by proton magnetic resonance spectroscopy.^[65]^ Brain lactate is the end product of anaerobic glycolysis, with its elevations as a result of decreased mitochondrial energy production.^[66]^ HBDH, known as a mitochondrial enzyme, reversibly reduces the free acetoacetate so produced into D-3-hydroxybutyrate.^[67,68]^ Emerging evidence showed that antidepressant treatments decrease inflammatory and improve mitochondrial dysfunction in patients with depression.^[69,70]^ Based on the above reasons, HBDH appears to change with mitochondrial dysfunction. Nevertheless, few studies have reported the relationship and mechanism between HBDH and depression, indicating the need for further studies.

### 4.6 Blood urea nitrogen

Urea is the end product of protein metabolism.^[71]^ This study by Hu et al found 10% of 260 hemodialysis patients had a diagnosis of depression using the Diagnostic and Statistical Manual of Mental Disorders, 4th edition and found those patients with lower monthly income, shorter duration of hemodialysis, and lower level of blood urea nitrogen were more likely to have a diagnosis of depression comparing patients with depression to those without depression. They considered that depression symptoms were usually associated with poor appetite and poor nutrition in hemodialysis patients with depression.^[72]^ Another study by Peng et al^[73]^ showed that biochemical parameters, such as urea nitrogen, alanine transaminase, uric acid, lactate dehydrogenase, and total protein, were significantly different between depression patients and healthy controls, and that multiple biochemical parameters in combination may improve the diagnostic effectiveness of depression and the complete management for depressive patients.

### 4.7 Uric acid

Researchers had shown that oxidative stress plays an important role in depression.^[74]^ Major antioxidative defenses include both enzymatic and nonenzymatic antioxidants.^[74]^ The enzymatic antioxidants levels, such as catalase, superoxide dismutase, and glutathione peroxidase, were modified in depressive patients.^[75]^ The levels of nonenzymatic antioxidants were also altered in depressive patients. In the body, albumin, uric acid, ascorbic acid, and bilirubin were some of the nonenzymatic antioxidants.^[76]^ Accumulating evidence showed reduction of these antioxidants in depressive patients.^[77,78]^ Moreover, SSRIs may modify the levels of both enzymatic antioxidants and the nonenzymatic antioxidants.^[79,80]^

Uric acid is a major contributor to total radical trapping capacity accounting between 38–47% of the entire total in contrast to vitamin C and vitamin E which contributes 13% to 17% and 2% to 8%, respectively.^[74]^ It was suggested that uric acid as an antioxidant could reduce oxidative damage to DNA by reacting with guanyl radical.^[74]^ The evaluation of the biochemical variable like uric acid is helpful for early diagnosis and monitoring of the treatment of depression.

### 4.8 Creatinine

Creatinine production derives from creatine and phosphocreatine metabolism; conversely, the creatine–phosphocreatine system plays an important role in cellular energy transport. The study by Zheng et al. indicated metabolites including lipid metabolism-related molecules, lipid/protein complexes, energy metabolism-related molecules (creatine, creatinine), and amino acids could help to discriminate depressive patients from healthy controls using a NMR-based metabonomic approach.^[81]^ In the above study, the investigator found the level of creatinine was significantly reduced in depressed patients, consistent with another study, in which urine was analyzed employing an ultraperformance liquid chromatography coupled to mass spectrometry method.^[82]^ Although the role of creatinine in depression is unclear, the foregoing results showed that creatinine may be referred to energy deficiencies associated with depression.

### 4.9 Cholesterol

The study by Stanojevic et al^[83]^ showed the levels of total cholesterol, low-density lipoprotein cholesterol, triglycérides and blood pressure were statistically significantly elevated in the depressive patients with metabolic syndrome compared with healthy controls. Moreover, in other studies, hypercholesterolemia was linked to the poor outcome of antidepressant therapy in depression.^[84–86]^ However, several other studies showed low cholesterol levels were also detected in depression.^[87,88]^ The previous findings were inconsistent, probably because of different study subjects, methods, or event, etc. Therefore, we should carry out further research to explore the role of cholesterol in the pathophysiology of depression.

### 4.10 Total protein

Peng et al^[73]^ found significant differences in total protein (TP), fasting blood glucose (FBG), alanine transaminase (ALT), lactate dehydrogenase (LDH), high-density lipoprotein-cholesterol (HDL-C), urea nitrogen (UN), uric acid (UA), creatinine (Cr), total bile acid (TBA), direct bilirubin (Dbil), indirect bilirubin (Ibil), total bilirubin (Tbil), and fructosamine (SF) between patients with depression and healthy controls. Moreover, multivariate analysis demonstrated that TP, UN, HDL-C, FBG, Tbil, SF, and Cr remained independently correlated with depression. Nevertheless, the exact mechanisms of the link between depression and low total protein remain unclear, and further studies are needed to explore the critical mechanisms involved in total Protein.

### 4.11 Pulse rate and respiration rate

Few studies have directly explored the relationship between pulse rate and respiration rate and depression. The study by Richards et al^[89]^ showed that lisdexamfetamine dimesylate (LDX) exhibited significant dose-response relationships for systolic blood pressure, diastolic blood pressure, and pulse and all vital sign endpoints tended to increase as LDX dose increased, with a linear relationship providing the best fit. In our study, the pulse rate of the comorbidity group was significantly higher than that of the hypertension group alone, which might be related to take antidepressants. A lot of factors had been considered to influence respiratory rates, like age, acute pain, temperature, fear, agitation, and shock.^[90]^ The study by Davies and Maconochie^[90]^ indicated body temperature was an independent determinant of respiratory rate in children and respiratory rate increased with the increase of body temperature. In another prospective study, the investigator showed psychological symptoms including anxiety and depression were the cause of dyspnea.^[91]^ The symptoms of increased respiratory rate, shortness of breath or altered depth were present for the dyspnea, which indirectly showed the association between depression and respiratory rate. Further studies are needed to detect the direct association between depression and pulse rate and respiratory rate.

Depression is a highly heterogeneous and common psychiatric disorder.^[69,70]^ In clinical practice, psychiatrists usually rely on subjective judgment and scale tests to diagnose depression. Early diagnosis of depression in patients with hypertension can be challenging to nonpsychiatrists, especially when the doctors lacked in experience of dealing with depression. Although many markers for depression had been identified previously, such as CRP,^[71]^ tumor necrosis factor-α (TNF-α),^[72]^ IL-6,^[73]^ the brain-derived neurotrophic factor (BDNF),^[74]^ and so on, the predictability of a single marker was poor. In this study, we utilized a large panel of markers to distinguish patients with comorbidity of hypertension and depression from those having hypertension only and determined which ones may be of value in predicting evolution of depression in patients with hypertension. This is one of the advantages of the present study. Another advantage is that the samples could be obtained with convenience, minimal invasiveness, and low cost. There are 3 limitations in our study. One is that it lacks in bioinformation complexity of subjects. Increasing such complexity can enhance the adaptation of SVM. Second, 2 groups of patients were chosen retrospectively and not consecutively, which might cause an enrollment bias and an erroneous classification by the algorithm. This is one of the main methodological limitations of our current study, which should be remedied in the future investigation using a prospective and consecutive design. The previous studies conducted by Singh et al and Jonsson et al showed low serum albumin levels have been associated with malnutrition.^[92,93]^ BMI is one of the parameters used to estimate nutritional status, and BMI < 20.5 kg/m^2^ is a risk cutoff for malnutrition.^[94]^ We reviewed the studies by Wang et al and Peng et al, and found that BMI was mentioned in the 2 studies.^[61,73]^ However, BMI did not differ significantly between patients with depression and those without groups in patients with chronic renal failure undergoing hemodialysis and between depressed patients and the control groups. In our data, BMI may have no statistical significance between patients having hypertension only and those having comorbidity of hypertension and depression. Additionally, inflammatory markers such as cytokines result in activating protein catabolism and malnutrition.^[95]^ A meta-analysis by Dowlati et al^[96]^ strengthened evidence that depression is accompanied by the activation of the inflammatory response system. Therefore, decreased BMI or malnutrition may be caused by depression itself. However, because of the absence of data on BMI in our study, we cannot draw any firm conclusion regarding whether BMI could affect the markers screening such as glucose, albumin, and blood urea nitrogen. The assessment of nutrition (at least for BMI) should be included in the analysis, which is the third limitation of our current study, and we should remedy the information in future studies. The side effects of antidepressant drugs include loss of appetite, nausea and other adverse reactions, which may bias markers screening. However, the dosage of all antidepressant drugs in West China Hospital was within the recommended range. Generally speaking, patients get used to the side effects of the medicine in a few days, and thus the biochemical indicators are unlikely to decrease. However, changes in biochemical indicators are inconsistent in previous studies after antidepressant treatment. One study showed that antidepressant treatment has increased serum albumin and blood urea nitrogen level;^[79]^ the other study showed serum albumin concentration did not change significantly before and after antidepressant treatment.^[95]^ This may suggest that in our study, depression per se and not antidepressant drug is associated with the change of biochemical indicators because appetite-related biochemical indicators did not increase or remained unchanged, but decreased instead. Unfortunately, we have no data available regarding the use of antidepressant drug, so that we cannot draw any firm conclusion. We will investigate and discuss the effect of antidepressant drugs in later work.

## 5 Conclusion

Support vector machine (SVM) can be applied to identify patients with comorbidity of hypertension and depression from those having hypertension. Such an approach has revealed a significant association between depression and blood tests and vital signs. It can facilitate clinical diagnosis of depression using commonly available laboratory markers. Further studies are needed to verify the role of these candidate markers for depression diagnosis.

## Author contributions

Data processing, manuscript preparation, and modification: Xiuli Song.

Data processing: Zhong Zhang.

Data acquisition: Rui Zhang, Miye Wang.

Study design, manuscript modification: Dongtao Lin, Tao Li, Junming Shao, Xiaohong Ma.

**Conceptualization:** Xiaohong Ma.

**Data curation:** Xiuli Song, Zhong Zhang, Junming Shao.

**Funding acquisition:** Xiaohong Ma.

**Methodology:** Zhong Zhang.

**Project administration:** Miye Wang.

**Resources:** Rui Zhang.

**Supervision:** Tao Li.

**Writing – original draft:** Xiuli Song.

**Writing – review & editing:** Dongtao Lin.
